# Role of leptin in the regulation of food intake in fasted mice

**DOI:** 10.1111/jcmm.15110

**Published:** 2020-03-15

**Authors:** Tong tong Ge, Xiao Xiao Yao, Feng Lian Zhao, Xiao han Zou, Wei Yang, Ran Ji Cui, Bing jin Li

**Affiliations:** ^1^ Jilin Provincial Key Laboratory on Molecular and Chemical Genetics The Second Hospital of Jilin University Changchun China

**Keywords:** baclofen, feeding behaviour, GABA‐B, hypothalamus, leptin

## Abstract

Leptin is well acknowledged as an anorexigenic hormone that plays an important role in feeding control. Hypothalamic GABA system plays a significant role in leptin regulation on feeding and metabolism control. However, the pharmacological relationship of leptin and GABA receptor is still obscure. Therefore, we investigated the effect of leptin or combined with baclofen on the food intake in fasted mice. We detected the changes in hypothalamic c‐Fos expression, hypothalamic TH, POMC and GAD67 expression, plasma insulin, POMC and GABA levels to demonstrate the mechanisms. We found that leptin inhibit fasting‐induced increased food intake and activated hypothalamic neurons. The inhibitory effect on food intake induced by leptin in fasted mice can be reversed by pretreatment with baclofen. Baclofen reversed leptin's inhibition on c‐Fos expression of PAMM in fasted mice. Therefore, these results indicate that leptin might inhibit fasting‐triggered activation of PVN neurons via presynaptic GABA synaptic functions which might be partially blocked by pharmacological activating GABA‐B. Our findings identify the role of leptin in the regulation of food intake.

## 1 INTRODUCTION

Feeding behaviour is one of the most basic physiological activities of humans and animals and is closely related to energy expenditure and metabolism. It is well acknowledged that acute or chronic fasting can trigger overeating and hyperphagia.^1^ In the past decades, leptin, a hormone encoded by *ob* gene and secreted by adipose cells, is well studied in energy balance and glucose metabolism.^2^, ^3^ A number of animal studies have shown that leptin can suppress appetite and reduce the food consumption via acting on specific neurons in hypothalamus.^4^

Hypothalamic gamma‐aminobutyric acid (GABA)ergic neurons play significant role in the control of feeding and metabolism. It has been shown that leptin‐responsive neurons in hypothalamus are mainly GABAergic and largely located in the dorsomedial hypothalamus (DM), the arcuate nucleus of hypothalamus (ARC) and the lateral hypothalamus (LH).^5^ It was reported that leptin can selectively activate anorexigenic pro‐opiomelanocortin (POMC) neurons via influencing presynaptic release of GABA.^6^ Recent electrophysiological study further confirmed that leptin can reduce inhibitory tone of POMC neurons via directly regulating presynaptic GABAergic neurons. Leptin injection prevented potentiation of spontaneous inhibitory postsynaptic current (sIPSC) in POMC neurons induced by 24 hours‐fasting treatment.^5^ There are two types of GABA receptors in the central nervous system (CNS), including GABA‐A and GABA‐B, both of which have been reported to have important effect in the regulation of feeding behaviour.^7^ It was found that administration of GABA‐A receptor agonist, muscimol, can enhance feeding behaviour.^8^, ^9^, ^10^, ^11^ Systematic administration of benzodiazepine partial receptor agonist provoked a hyperphagic response.^9^, ^12^ Conversely, some evidence reported that administration of GABA‐A antagonist into the LH promote eating, while the agonist of GABA‐A receptor suppressed feeding.^13^ Intracerebroventricular administration of antagonist of GABA‐A receptor bicuculline increased eating and drinking behaviours.^14^ Pharmacological studies showed that peripheral or intracranial administration of the GABA‐B agonist baclofen increased food intake.^15^, ^16^, ^17^, ^18^, ^19^ Conversely, administration of GABA‐B receptor antagonist 3‐aminopropyl‐diethoxy‐methyl‐phosphinic acid (CGP 35348) reduced food consumption in rats after 22 hours of fasting.^20^ Chronic systematic administration of 4 mg/kg baclofen did not affect daily cumulative food intake, but reducing the bodyweight gain in comparison with control rats.^21^ These results indicate that GABA receptors exert potential anti‐anorexia effect via different molecular mechanisms. A recent research found that leptin‐mediated inhibition of orexin neuron activities was independent of GABA‐A receptor.^22^ Currently, still few studies investigated the pharmacological effect of GABA‐B receptor implicated in the anorectic effects of leptin. Therefore, in the present study, we investigated the role of GABA‐B receptor played in the feeding regulation of leptin in fasted mice and non‐fasted mice. We further demonstrate the potential molecular mechanisms via investigating hypothalamic neuronal activation, glutamate decarboxylase 67 (GAD67), tyrosine hydroxylase (TH) and POMC protein levels of hypothalamus, serum insulin, POMC and GABA levels alterations after systematic administration of leptin or combined with baclofen.

## 2 MATERIALS AND METHODS

### 2.1 Animals

All animals we used were 5‐week‐old, 20 ± 2 g weight imprinting control region (ICR) strain male mice which were purchased from Jilin University. All mice were single‐housed in a cage (25.5 × 15 × 14 cm) for at least 1 week before all experimental procedures. This study was carried out under the approval from the ethics committee of Second Hospital of Jilin University.

### 2.2 Drugs

Leptin (Tocris) was dissolved in saline in dose of 1 mg/kg. About 1 mg leptin was dissolved in 1 mL saline, and the compound was further diluted into final concentration of 0.1 mg/mL for injection. Baclofen (Sigma) was dissolved in saline in dose of 8 mg/kg. About 80 mg baclofen was dissolved in 10 mL saline, and the compound was further diluted into final concentration of 0.8 mg/mL for injection. All drugs and vehicle (saline) were intraperitoneally (i.p.) injected in a volume calculated based on the bodyweight (0.01 mL/g).

### 2.3 Food intake measurements

Before food intake measurements, each animal was single‐housed and fasted for 16 hours (overnight) from 5:00 pm to the next day 9:00 am. Animals were injected with leptin (1 mg/kg), baclofen (8 mg/kg) and saline according to the experimental groups. Baclofen was injected 15 minutes before leptin injection. After all drug treatment, each animal was placed in a new cage and refed with 4‐6 food pellets. The food intake was measured at 0.5, 1 and 1.5 hours after the final injection as we described previously.^23^ For calculation of the cumulative food intake, the latter weight of feed was subtracted from the former value. After recording the food intake, all mice were killed by decapitation. The brain tissue and blood sample were saved for subsequent neurochemical test.

### 2.4 Western blot analysis for GAD67, TH and POMC

Hypothalamus were extracted from saved brain tissue and homogenized with lysis buffer (137 mmol/L NaCl, 20 mmol/L TRIS, 1% NP40, 10% glycerol, 1 phenylmethylsulphonyl fluoride (PMSF), 10 μg/mL aprotinin, 1 μg/mL leupeptin, 0.5 mmol/L sodium vanadate, 0.5 mmol/L sodium fluoride) on ice. The lysates were then further centrifuged and boiled. Western blot method was same as our previous studies.^23^ The samples were resolved by 10% sodium dodecyl sulfate—polyacrylamide gel electrophoresis (SDS‐PAGE) and transferred to polyvinylidene difluoride (PVDF) membranes, which were further blocked with 5% skim milk. The membranes were incubated with diluent of anti‐POMC (Abcam, ab32893; 1:1000), anti‐TH (Millipore Sigma, ab152, 1:2000), β‐Actin (TransGen Biotech, #HC201, 1:2000) and anti‐GAD67 (Abcam, ab26116, 1:1000) overnight at 4°C. After three times of washing with Tris‐buffered saline with Tween 20 buffer, membranes were then incubated in horseradish peroxidase (HRP)‐conjugated secondary antibodies (Proteintech, SA00001‐2, 1:1000), and then membranes were exposed to chemiluminescent detection reagents and imaged. The optical density of all sample bands was detected by ImageJ software.

### 2.5 c‐Fos immunohistochemistry

c‐Fos immunohistochemistry analysis method was presented as our previous studies.^23^ All mice were intraperitoneal injected with 400 mg/kg chloral hydrate for anesthetization. The perfused fixed brain tissue was cut into 30 μm slices on a cryostat. After washing three times in phosphate buffer saline (PBS) containing 0.1% Triton X‐100 (PBST), brain slices were incubated 15 minutes with 0.6% hydrogen peroxide in PBS. After three times washing with PBS, sections were incubated with anti‐c‐Fos (Santa Cruz, CA; #sc‐52, 1:1000) primary diluents which is diluted with PBS containing 0.3% Triton X‐100, 0.05% sodium azide and 2% normal goat serum for 72 hours at 4°C. The sections were next successively incubated with biotinylated secondary antibody diluent (ZSGB‐Bio, ZB2305, 1:400) and the diluent of avidin‐biotinylated horseradish peroxidase complex for 75 minutes at room temperature, and then using the glucose oxidase‐diaminobenzidine‐nickel method to visualize the reaction product and subsequently terminating the reaction by washing in sodium acetate buffer. Sections were attached onto chrome alum/gelatin‐coated slides and air‐dried at room temperature. The c‐Fos‐positive cells were identified by dense brown nuclear staining and counted under ×200 magnification from different nucleus of hypothalamus. The c‐Fos‐positive neurons of per bilateral regions were pictured under microscopy (Eclipse 50i Microscope, Nikon) at least from 5‐8 sections as described previously.^24^ The counting was based on average of counting c‐Fos numbers by experimenters who were blind to the experimental conditions.

### 2.6 ELISA analysis for plasma insulin, GABA and POMC

Collected blood was left at 4°C overnight and then centrifuged at 4°C for 15 minutes at 1509 *g*. And then, we transferred the serum to a new eppendorf tubes until use. We use Mouse GABA, insulin and POMC (Jiancheng) ELISA Kits to detect the serum insulin, GABA and POMC levels according to the manufacturers' instructions.

### 2.7 Statistical analysis

All data are presented as mean ± SEM Statistical analysis was performed with SPSS statistics for Windows (version 25.0). All data we presented were using one‐way analysis of variance (ANOVA) followed by a post hoc Dunnett's test. Significant differences were considered exist when the *P* values were less than .05.

## 3 RESULTS

### 3.1 Effect of leptin or combined with baclofen on food intake in fasted mice

**Figure 1:**
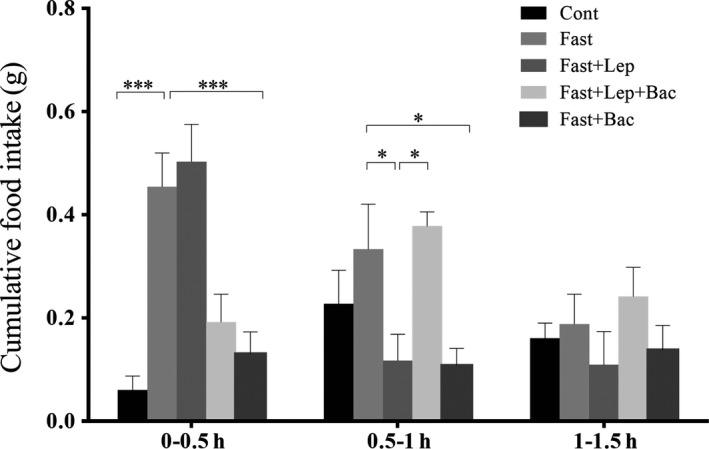
Effect of leptin or combined with baclofen on cumulative food intake in fasted mice. Cont: control group; Lep: Leptin (1 mg/kg); Bac: Baclofen (8 mg/kg); Columns represent the mean ± SEM n = 8. (*P < .05, **P < .01, ***P < .001, n = 8 mice per group)

Firstly, we detected the cumulative food intake in the 0‐1.5 hours period after all drug treatments (Figure 1). We found that 16 hours food deprivation significantly increased the food intake during 0‐0.5 hours (one‐way ANOVA, *F*_(4,35)_ = 15,755, *P* < .001). We found that pretreatment with 8 mg/kg baclofen significantly reduce the food intake during 0‐0.5 hours period (*P* < .001) and 0.5‐1 hours (*P* < .05). During the 0.5‐1 hours period after drug treatment (one‐way ANOVA, *F*_(4,35)_ = 5.457), we found that leptin treatment significantly reduced food intake in comparison with the fasting group (*P* < .05), and baclofen pretreatment reversed this effect (*P* < .05). No significant difference on cumulative food intake was detected in 1‐1.5 hours period after drug treatment (one‐way ANOVA, *F*_(4,35)_ = 1.055).

### 3.2 Effect of leptin or combined with baclofen on c‐Fos expression in hypothalamus of fasted mice

**Figure 2:**
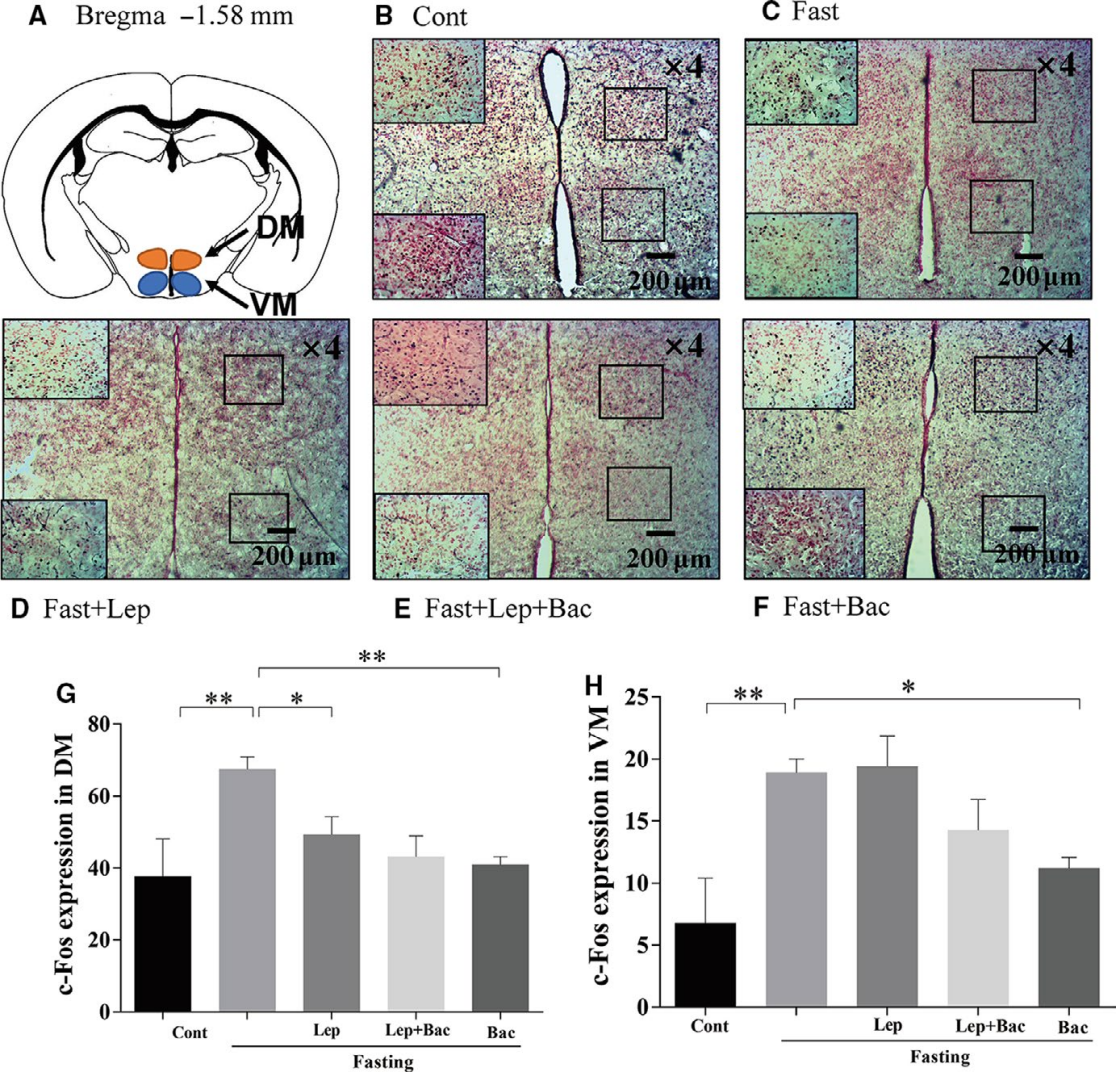
Effect of leptin or combined with baclofen on c‐Fos expression in hypothalamus. A, Schematic image of the DM and VM of hypothalamus from mouse brain stereotaxic coordinates. B‐F, Representative photomicrographs of c‐Fos expression in the DM and VM of different groups. G, H, Quantitative analysis of the c‐Fos expression in DM and VM; DM: dorsal medial nucleus of hypothalamus, VM: ventromedial nucleus of the hypothalamus; Data are represented as the mean ± SEM; (*P < .05, **P < .01. n = 2‐3 mice per group)

**Figure 3:**
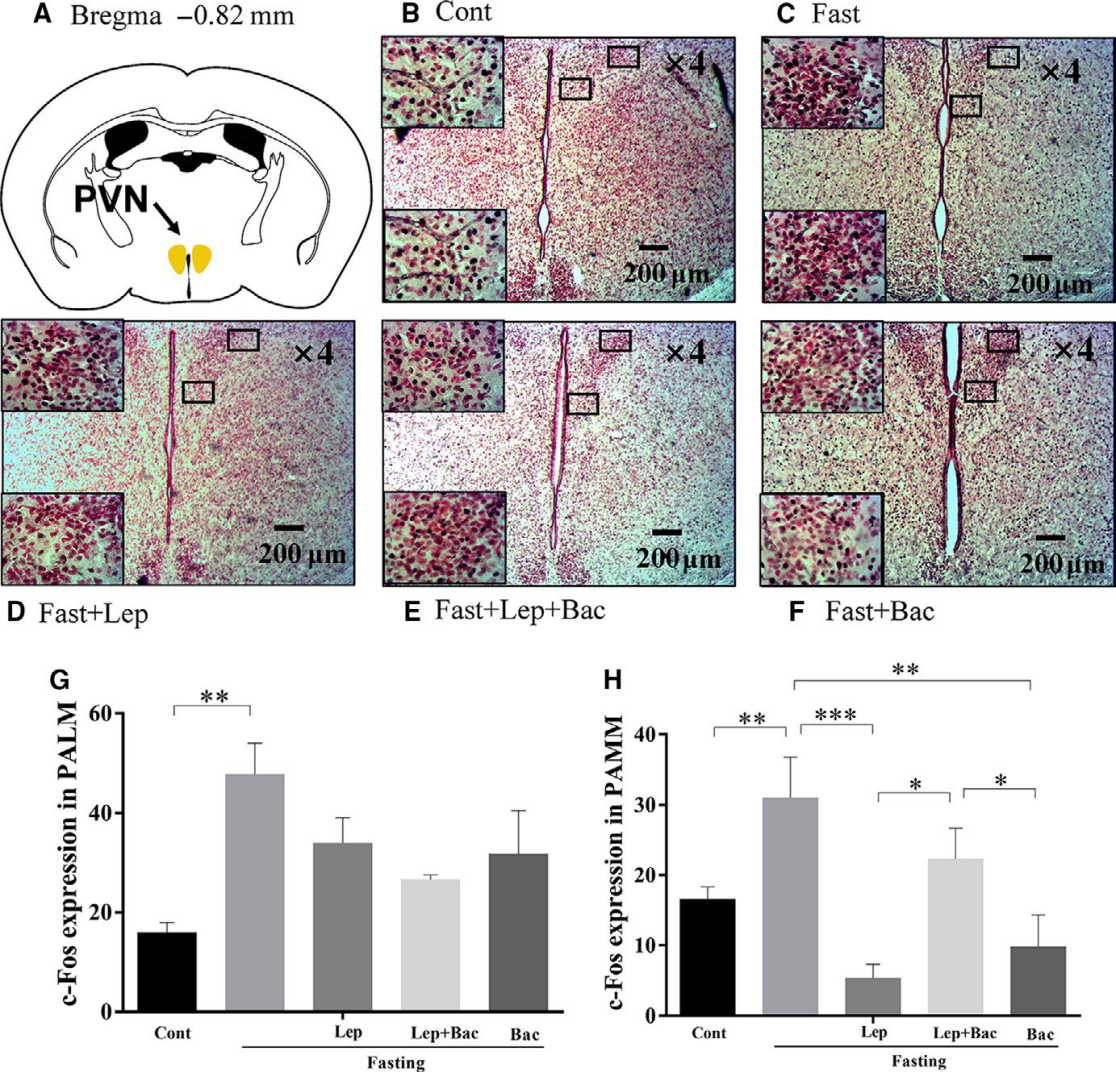
Effect of leptin or combined with baclofen on c‐Fos expression in PVN of hypothalamus. A, Schematic image of the PVN from mouse brain stereotaxic coordinates; B‐F, Representative photomicrographs of c‐Fos expression in the PAMM and PALM of PVN in different groups. G, H, Quantitative analysis of the c‐Fos expression in the PAMM and PALM of PVN in different groups; PVN: paraventricular thalamus of hypothalamus; PAMM: medial magnocellular part of paraventricular hypothalamic nucleus; PALM: lateral magnocellular part of paraventricular hypothalamic nucleus; Data are represented as the mean ± SEM; (*P < .05, **P < .01, ***P < .001. n = 2‐3 mice per group)

**Figure 4:**
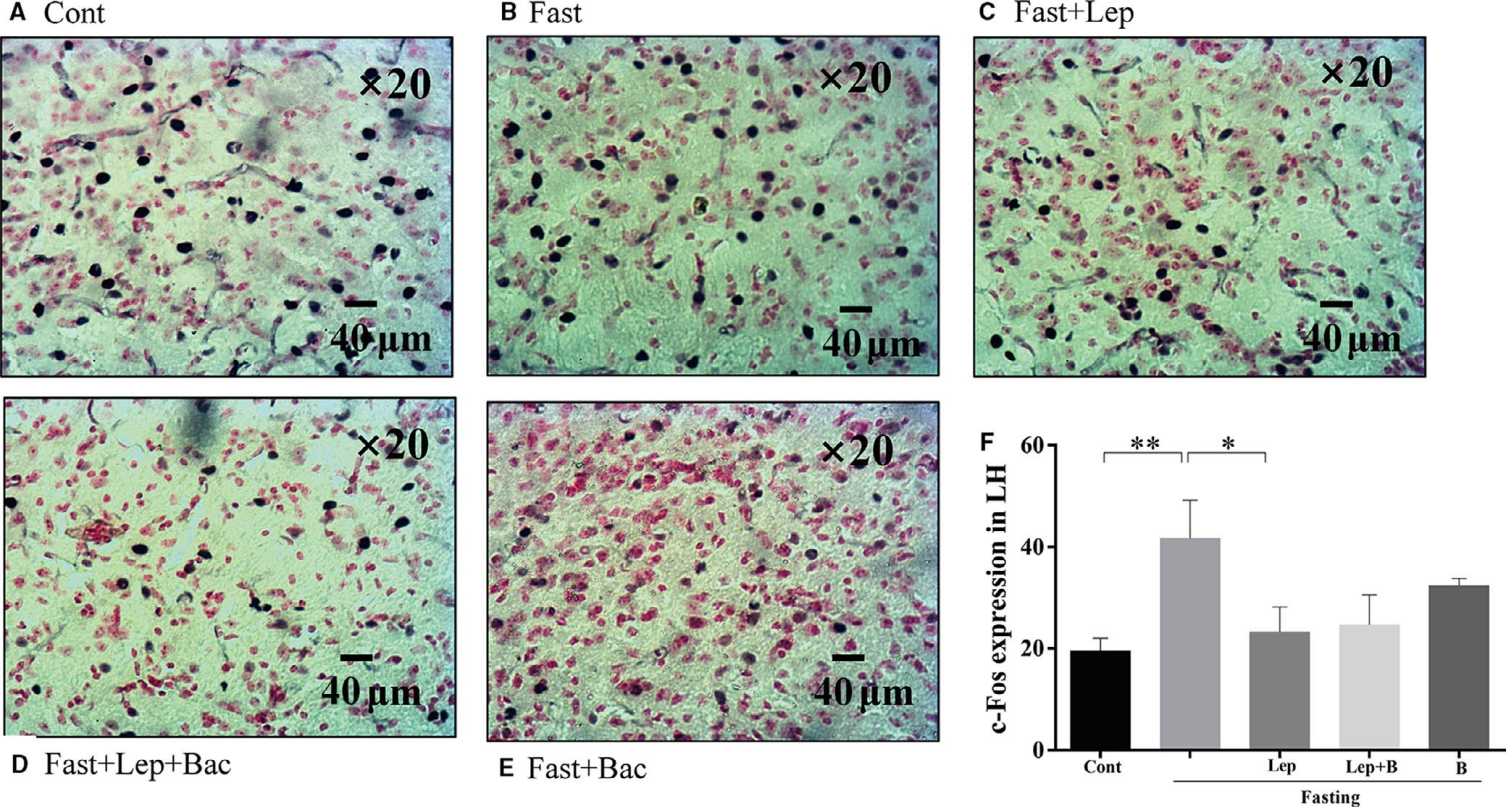
Effect of leptin or combined with baclofen on c‐Fos expression in hypothalamus. A‐E, Representative photomicrographs of c‐Fos expression in the LH; F, Quantitative analysis of the c‐Fos expression in the LH. LH: lateral hypothalamus; Data are represented as the mean ± SEM; (*P < .05, **P < .01, ***P < .001. n = 2‐3 mice per group)

**Figure 5:**
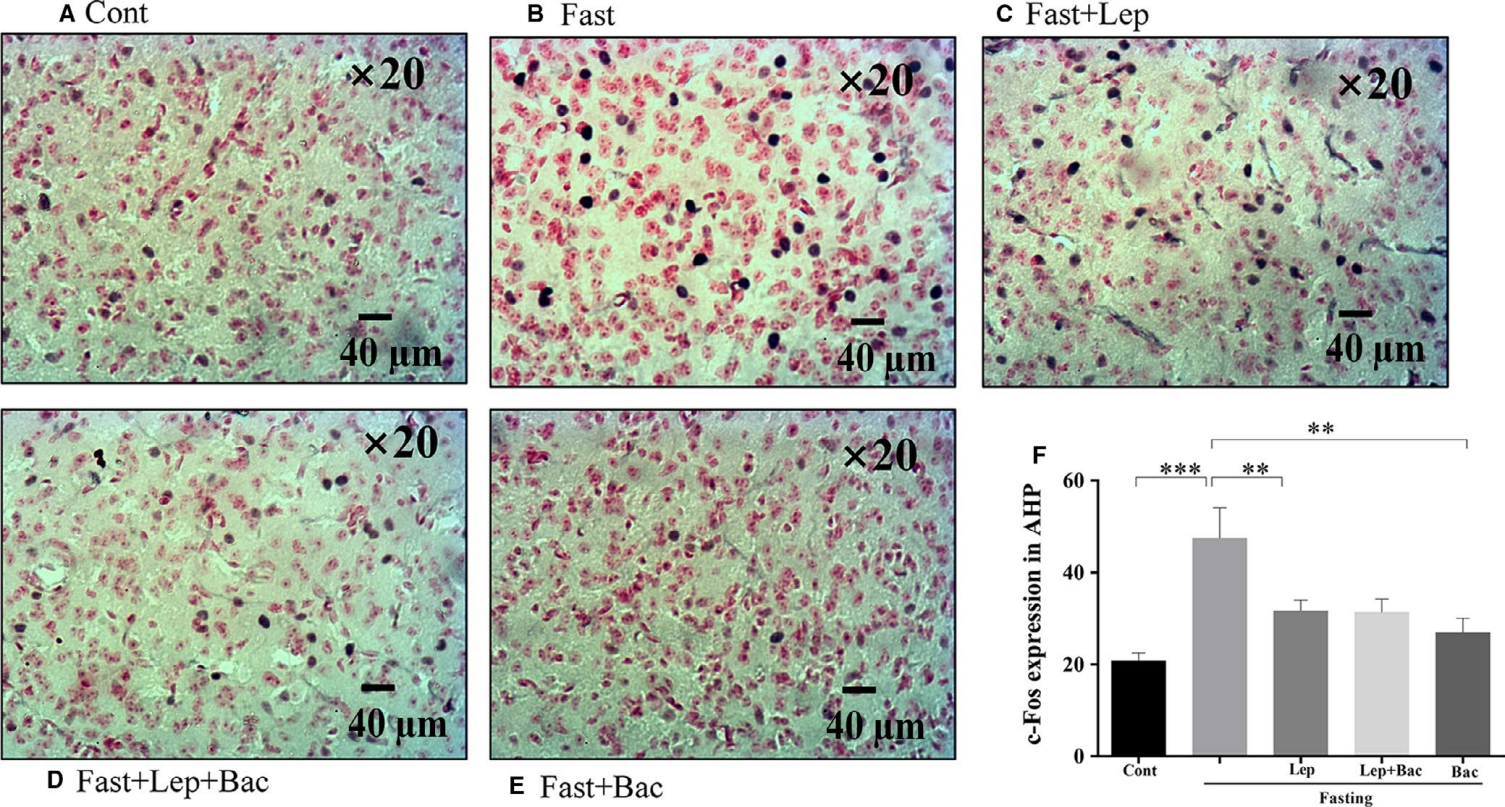
Effect of leptin or combined with baclofen on c‐Fos expression in hypothalamus. A‐E, Representative photomicrographs of c‐Fos expression in the AHP; F, Quantitative analysis of the c‐Fos expression in the AHP; AHP: anterior hypothalamic nucleus; Data are represented as the mean ± SEM; (*P < .05, **P < .01, ***P < .001. n = 2‐3 mice per group)

We next detected the neuronal activation of hypothalamus in fasted mice after leptin or combined with baclofen treatment. We found that 16 hours food deprivation significantly increased c‐Fos expression in DM (Figure 2G, *F*_(4,19)_ = 4.489), ventromedial nucleus of the hypothalamus (VM) (Figure 2H, *F*_(4,18)_ = 5.863), lateral magnocellular part of paraventricular hypothalamic nucleus (PALM) (Figure 3G, *F*_(4,18)_ = 4.085), medial magnocellular part of paraventricular hypothalamic nucleus (PAMM) (Figure 3H, *F*_(4,19)_ = 6.566), LH (Figure 4F, *F*_(4,20)_ = 3.174), and anterior hypothalamic nucleus (AHP) (Figure 4F, *F*_(4,20)_ = 7.001). Systematic injection of leptin reduced the c‐Fos expression of DM (*P* < .05), PAMM (*P* < .001), LH (*P* < .05) and AHP (*P* < .01, Lep group versus Fast group). Pretreatment with baclofen reversed the reduction of c‐Fos expression induced by leptin in PAMM of hypothalamus (*P* < .05, Lep group versus Lep + Bac group). We also found that single baclofen injection after fasting significantly reduced the c‐Fos number in DM (*P* < .01), VM (*P* < .05), PAMM (*P* < .01) and AHP (*P* < .01) of hypothalamus (Bac group versus Fast group. Figure 5).

### 3.3 Effect of leptin or combined with baclofen on GAD67, TH and POMC expression in fasted mice

**Figure 6:**
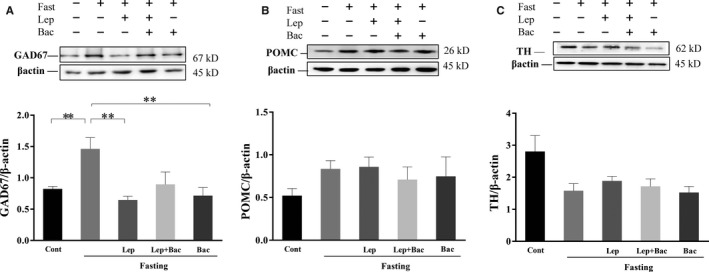
Effect of leptin or combined with baclofen on GAD67, POMC and TH expression in hypothalamus. A, Representative Western blots for GAD67 and β‐actin. B, Representative Western blots for POMC and β‐actin. C, Representative Western blots for TH and β‐actin. Data are represented as the mean ± SEM; (*P < .05, **P < .01, ***P < .001. n = 5‐6 mice per group)

To further investigate the effect of leptin and baclofen administration on expression of appetite‐related peptides, synthesis of GABA and dopamine (DA) in hypothalamus of fasted mice, we used Western blot measurement to detect the expression of GAD67 (Figure 6A, *F*_(4,20)_ = 5.408), TH (Figure 6B, *F*_(4,20)_ = 3.262) and POMC (Figure 6C, *F*_(4,20)_ = 0.807) in protein levels. We found that 16 hours fasting significantly increased the GAD67 expression (*P* < .01, versus Cont group). Systematic injection of leptin reduced GAD67 expression (*P* < .01, Lep group versus Fast group). Baclofen administration alone also decreased GAD67 expression (*P* < .01, Bac group versus Fast group). We observed increased TH expression in fasted mice (*P* < .05, Fast group versus Cont group). There was no significant difference in POMC expression between groups.

### 3.4 Effect of leptin or combined with baclofen on insulin POMC and GABA levels in fasted mice

**Figure 7:**
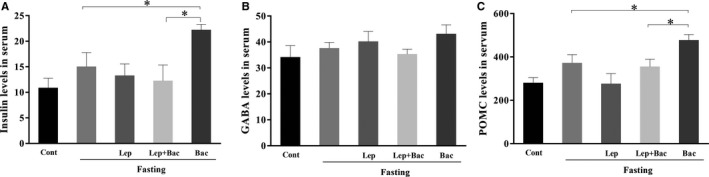
Effect of leptin or combined with baclofen on plasma insulin, GABA and POMC levels. A, Effect of leptin or combined with baclofen on plasma insulin levels. B, A, Effect of leptin or combined with baclofen on plasma GABA levels. C, Effect of leptin or combined with baclofen on plasma POMC levels. Data are represented as the mean ± SEM; (*P < .05, **P < .01, ***P < .001. n = 5‐6 mice per group)

We finally detected serum insulin (Figure 7A, *F*_(4,29)_ = 3.556), GABA (Figure 7B, *F*_(4,29)_ = 1.182) and POMC (Figure 7C, *F*_(4,25)_ = 5.503) levels of fasted mice after drug treatment. ELISA analysis results showed that the serum insulin levels of mice treated with baclofen were significantly higher than the Fast group (*P* < .05). Baclofen treatment also enhanced serum POMC levels (*P* < .05, Bac group versus Fast group). We also observed a mild increment of serum leptin levels after 16 hours fasting (*P* < .05, Fast group versus Cont group). There was no significant difference between other groups.

## 4 DISCUSSION

Our result found that 16‐hour food deprivation significantly increased the cumulative food intake and the increase was gradually weakened over time as previously reported.^25^ Systematic injection of 1 mg/kg leptin inhibited fasting‐induced increase of food consumption in mice. This action of leptin was the most significant in the 0.5‐1 hours period after drug treatments. We also observed that pretreatment with baclofen reversed leptin‐induced reduction of food intake in fasted mice, indicating that GABA‐B receptor might be implicated in the regulation of food intake by leptin. We have previously reported that 5‐HT_3_ receptor agonist SR‐57227 significantly inhibited the food intake of fasted mice which can be blocked by 5‐HT_3_ receptor antagonist ondansetron.^26^ Some evidence have shown that systematic baclofen administration can increase the food intake of animals in a dose‐dependent manner.^16^, ^27^, ^28^ Chronic 2 mg/kg baclofen treatment enhanced short‐term food intake while had no significant effect on daily cumulative food intake and the weight gain of rats.^19^ Ebenezer et al^27^ reported that 1‐8 mg/kg systematic administration of baclofen caused a dose‐related increase in cumulative food intake and the increase was the most significant in CFLP mice treated with 8 mg/kg baclofen. They subsequently reported that the promotion of baclofen on food intake was related to hunger or satiety conditions and 1‐4 mg/kg baclofen treatment had no significant effect on feeding behaviours of rats treated with 22‐hour food deprivation,^28^ which is consistent with an earlier study.^29^ 1‐4 mg/kg baclofen can increase food intake of 16‐hour fasting rats only in the first 30 minutes after drug administration.^28^ However, no investigations demonstrated the effect of 8 mg/kg baclofen treatment on feeding behaviour after 16 hours food deprivation. Therefore, in the present study, we choose 8 mg/kg baclofen administrated to the fasted mice and to further investigate the role of leptin in feeding regulation of fasted mice. Intriguingly, inconsistent with these results, we found that 8 mg/kg baclofen alone cause a significant reduction of food intake within 0‐1 hour period after 16‐hour food deprivation. These results suggest that the effect of baclofen on feeding behaviour might be related to the dosage of administration of baclofen and the energy states of animals.

Some evidence demonstrated that most leptin‐responsive neurons are GABAergic and mainly located in LH, DM and ARC.^5^, ^30^ In addition, paraventricular thalamus of hypothalamus (PVN) neurons play critical role in leptin action in hypothalamus as PVN can receive vast of GABAergic projections from DM, LH and ARC.^31^ Fasting can induce transcriptional alterations in PVN and ARC, decrease POMC levels and promote agouti gene‐related protein (AgRP) levels, and these alterations were shown to relate to fasting‐induced leptin fall and could be partially reversed by systematic treatment of leptin.^32^, ^33^, ^34^, ^35^Consistent with that, in our findings, we observed that leptin cause a reduction of fasting‐induced increased expression of c‐Fos in LH and DM, PAMM, AHP, while not in the VM, these findings were consistent with our previous work.^26^

Hypothalamic GABAergic neurons have been shown to play an important role in feeding control.^36^ Inhibition of GABAergic neurons reduce food consumption, conversely inhibition of glutamatergic neurons enhance feeding.^4^ A recent work has demonstrated that leptin can indirectly reduce of inhibitory tone to postsynaptic POMC neurons by activating presynaptic GABAergic neurons. They further found that systematic injection of 4 mg/kg leptin can reverse 24 hours fasting‐induced potentiation of frequency and amplitude of sIPSC in POMC neurons.^5^ Leptin receptor (LepR)‐expressed GABAergic neurons in DM inhibit fasting‐promoted AgRP via potentiation of presynaptic GABA release.^37^ Leptin can reverse fasting‐induced activation of the PVN neurons expressed melanocortin‐4 receptor (MC4R), which can be activated by POMC‐derived neuropeptides.^38^ In addition, it has been found that disruption of GABA release from adult LH neurons can reduce monosynaptic IPSCs in PVN neurons via LH‐PVN GABAergic projections and potently reduce feeding.^39^ These results indicate that GABAergic microcircuitry in hypothalamus might mediate activation or inhibition of POMC and AgRP in response to different energy state. Our observation that pretreatment with baclofen blocked leptin‐induced reduction of c‐Fos expression only in PAMM nucleus under fasting conditions. It might be due to PVN can receive projections from leptin‐responsive neurons located in other hypothalamic regions, such as the ARC, DM and LH. Leptin might indirectly or directly regulate activation of PVN neurons via modulating GABAergic connections from other regions of hypothalamus. The inhibition of leptin on fasting‐induced overactivities in PAMM might be partially blocked by pharmacological activation of GABA‐B via systematic baclofen injection. We also observed higher GAD67 expression in hypothalamus of fasted mice which was inhibited by leptin. These results suggest leptin might inhibit the GABA synthesis induced by fasting. Although we did not detect significant difference in serum GABA levels, low sensitivities of neurotransmitters in serum samples might give an explanation. Thus, the specific mechanisms of GABA‐B receptor implication in leptin‐mediated feeding control still need further investigations.

Leptin has been shown to modulate mesolimbic DA system via activating LepR‐containing LH neurons.^40^ Earlier research reported fasting can promote expression of TH, a catecholamine biosynthesis‐mediated enzyme, and DA release.^41^, ^42^ However, in present study, we observed a reduction of TH expression of hypothalamus in fasted mice, indicating that the role of catecholaminergic neurons played in fasting‐induced overeating needs to be further investigated. It was reported that LepR‐expressed GABA and POMC neurons were implicated in increased glucose utilization upon insulin deficiency.^30^ However, we did not observe statistical difference of insulin levels after leptin or combined with baclofen injection in fasted mice. We found that 8 mg/kg baclofen injection significantly increased serum insulin levels, indicating that GABA system might be synergic with insulin regulation in dietary metabolic.

In addition, some pharmacological evidence showed the role of GABA‐A receptor played in feeding control. Infusion of a nonspecific GABA‐A receptor agonist muscimol to PVN elicit feeding behaviour.^11^ However, some findings showed that administration of GABA‐A antagonist into the LH promoted eating, while the agonist of GABA‐A receptor suppressed feeding.^13^ Intracerebroventricular administration of antagonist of GABA‐A receptor bicuculline increased eating and drinking behaviours.^14^ Systematic administration of benzodiazepine partial receptor agonist provoked a hyperphagic response.^9^, ^12^ Loss of GABA‐A receptor function of the PVN disrupted neuropeptide Y (NPY)‐induced hyperphagia, which depend on GABAergic projections from non‐POMC non‐AgRP neurons.^43^ Similarly, deletion of GABA‐A receptor in the PVN reduced feeding.^39^ However, few studies demonstrated the role of GABA‐B played in hypothalamus feeding control.

Some limitations still exist in the present article. Firstly, do not reconcile with previous research showing significant alterations of POMC expression induced by leptin treatment or food deprivation, and we did not find significant changes of POMC expression in Western blot measurement and ELISA test. We propose that refeeding process after drug administration before killing might cause compensate expression of anorectic peptides. Secondly, our strategy still lacks in comparative experiments and analysis on GABA‐A and GABA‐B receptor and other antagonists or agonists of GABA‐B receptor. Finally, recent findings have been shown that leptin signalling of astrocytes in hypothalamus also implicates in feeding regulation.^27^ Thus, comprehensive consideration of the interactions of hormones, synaptic connections with different type cells in hypothalamus is important to understand leptin action in feeding control. These issues need to be further investigated.

In summary, these findings indicate that leptin inhibit fasting‐promoted food intake and activation of hypothalamic neurons. The inhibition of leptin on food intake of fasted mice can be prevented by pretreatment with GABA‐B receptor agonist baclofen. We speculate that leptin might inhibit fasting‐triggered activation of PAMM neurons via presynaptic GABA synaptic functions and this effect might be partially blocked by pharmacological activating GABA‐B. The specific neurophysiological mechanisms of GABA implicated in the role of leptin played in feeding regulation of fasted mice need to be further investigated.

## CONFLICT OF INTEREST

The authors declare that the research was conducted in the absence of any commercial or financial relationships that could be construed as a potential conflict of interest.

## AUTHOR CONTRIBUTIONS

TTG, FLZ, XXY and XHZ conceived the idea and performed experiments. WY, RJC and BJL wrote the manuscript. BJL revised the manuscript.

## Data Availability

Data are available on request.
